# Anxiety and Coping Strategies during the Covid-19 Pandemic among Students at a Multi- Campus University in Uganda

**DOI:** 10.21203/rs.3.rs-1446865/v1

**Published:** 2022-03-29

**Authors:** Daniel Ojilong, Andrew Marvin Kanyike, Ashley Winfred Nakawuki, Dinah Maria Lutwama, Dorothy Nakanwagi, Rebecca Nekaka

**Affiliations:** Busitema University; Busitema University; Busitema University; Busitema University; Busitema University; Busitema University

**Keywords:** Anxiety, Coping, COVID-19, Students, school closure, university, Uganda

## Abstract

**Background:**

Uganda has had the longest COVID-19-induced closures of schools world over of over 20 months, according to a recent UNICEF report, which has greatly affected learning and mental health of University students. This study assessed levels of anxiety, challenges and coping strategies of students at a university in Uganda during the COVID-19 pandemic lock down.

**Methods:**

We conducted an online, descriptive, cross-sectional study between 26th June and 26th July 2021 using mixed quantitative and qualitative methods among students of Busitema University in Eastern Uganda. The survey assessed anxiety levels of students using General Anxiety Disorder 7 (GAD-7) scale, and its associations using the Chi-Square or Fischer’s exact test and multivariate logistic regression. We also explored the challenges and coping strategies employed by students through in-depth interviews.

**Results:**

A total of 338 students participated, 213 (63%) were male with median age of 23 years (21–25), majority from Faculty of health sciences (n = 153, 45%). Overall, 179 (53%) of the students had anxiety which was mostly mild anxiety (n = 127, 38%). Students concerned about inadequate internet facilities to support online learning were twice more likely to have anxiety (aOR 2.0, 95% CI 1.1–3.7; p = 0.021). Among those with anxiety, avoidance coping strategies had higher scores with a median of 8 (3–12) compared to other strategies (p < 0.001). In-depth interviews revealed challenges with online learning, academic progress, and changes to daily routine and fear of contracting COVID-19 and getting vaccinated.

**Conclusion:**

The largest number of students had anxiety especially those from faculty of health sciences and engineering of which most used avoidance strategies to cope up with the anxiety. This highlights areas where the university authorities should gear effort to design appropriate strategies to maintain mental health of students even after the pandemic.

## Background

Corona Virus Disease 2019 (COVID-19) continues to create mayhem around the globe [1]. Countries instituted restrictive public health measures to curb its spread which included among others; closure of borders and public transport, social distancing with stay home orders, and closure of schools [4]. Uganda has had the longest COVID-19-induced closures of schools world over of over 20 months, which has greatly affected learning and mental health of University students. These restrictions have enormously increased levels of psychological distress (anxiety, stress and depression) among different people [5,6].

Anxiety is highly prevalent among students in higher institutions of learning of which the top three concerns are academic performance, pressure to succeed and post-graduation plans [7]. Additionally, heavy course loads, stringent examinations, continued pressure to attain a high grade point average [8], complex interpersonal relationships, challenges of the clinical environment [9] result in greater anxiety among medical students than those in other disciplines. Anxiety has a negative effect on the quality of students’ life, their education [10] and may cause drop out from the programs.

Amidst the pandemic state, university students are even exposed to additional stressful factors such as fear of being infected. A study conducted among nursing students during the Severe Acute Respiratory Syndrome (SARS) outbreak (2003) in Hong-Kong showed that they perceived themselves to be at higher risk of infection [11]. Similarly, from studies done during the Middle East Respiratory Syndrome (MERS) outbreak (2016), high stress levels among healthcare students were associated with reluctance to work in healthcare facilities and providing care for patients [12,13]. University life and learning environment play a vital role in the psychological development of students and home confinement has been hypothesized to have a psychological impact [14]. In a study among Chinese college students, they reported that undergoing confinement made them feel deprived of their liberty, and felt bored with being on their own, which was associated with negative psychological impact [15]. Among other factors that have been associated with higher anxiety levels in students are low knowledge and belief in conspiracy theories surrounding the pandemic [16]. Coping with anxiety and stress maybe challenging to students and a study showed better coping strategies among nurses using problem-focused methods compared to nursing students who choose immature or negative coping strategies [17].

In Uganda a nation-wide mandatory closure of all institutions of learning was imposed at around mid-March 2020 and an isolation policy was introduced by the government with only about 25% of workers in both public and private sectors being allowed to continue working [18]. The first lockdown was eased and learning institutions opened in February 2021 however they were shortly closed again after four months due to a more deadly second wave of the pandemic. Both staff and students of academic institutions faced a new reality of shifting to online teaching which is faced with a number of challenges including poor network connectivity and high costs of internet. However amidst this uncertainty of learning continuity, the mental status of students remains unknown. Therefore this study assessed levels of anxiety, challenges and coping strategies of students at a university in Uganda during the second covid-19 pandemic lock down.

## Method

### Study design

We conducted a descriptive, cross-sectional study between 26th June and 26th July 2021 using both quantitative and qualitative methods.

### Study setting

The study was carried out among students of Busitema University which is a multi-campus model public University located in the Eastern region of Uganda with an estimated population of 4,052 students as of 2018/2019 intake. The Faculty of engineering with 710 is located in Tororo town, Faculty of science and education 774 in Nangogera, Faculty of natural resources and environmental sciences 116 in Namasagali, Faculty of Agriculture and animal sciences 1768 in Sororti city, Faculty of health sciences 517 in Mbale city, and Faculty of management sciences 167 students in Pallisa town. The university was established in 2007 and it focuses on relevant and critical study programs in Engineering, Science Education, Health Sciences, Natural Resources and Environmental Sciences, Agriculture, Animal Sciences, and Management Sciences. It currently has six operational campuses and offers 44 academic programs across various disciplines

### Study population

All students at Busitema University across the six campuses pursuing different academic disciplines

### Inclusion and exclusion criteria

Individuals aged 18 years or older, students at Busitema University who consented to participate were included and those students who couldn’t access internet were excluded.

### Data collection tool

#### Quantitative tool

The questionnaire was adapted from Nurunnabi et al [19] and Barolan et al [20] with modifications to suit our study. It had 3 parts, the first obtaining demographic data and the second, level of anxiety using the General Anxiety Disorder 7 item scale with each item coded from zero to three, per frequency of different anxiety symptoms and the total score was computed by summing up the responses. Categorizations of the GAD-7 scores was used as per the original scale (i.e. 0–4 = minimal anxiety, 5–9 = mild anxiety, 10–14 = moderate anxiety, and > 14 = severe anxiety). A score of 10 and above was taken as diagnostic of GAD with a sensitivity of 89% and a specificity of 82%. The coping strategies were assessed from four broad categories: “Seek social support,” “Avoidance,” “Mental disengagement” and “Humanitarian”. The items were measured on a scale of 1 to 4 (1 = never/rarely, 2 = sometimes, 3 = often and 4 = very often/always).

#### Qualitative tool

For the in-depth interview, an interview grid was used. This included open ended questions followed by probing points targeting specific pandemic stressors derived from existing literature identifying prominent factors affecting university students’ mental health [21].

### Sampling Procedure and Data collection

#### Quantitative Data

During this study, Uganda was in the second lockdown with schools, universities, and institutions closed conducting ODEL (Open Distance Electonic-Learning). Therefore, we opted to use WhatsApp Messenger (Meta Inc) for enrolling potential participants. We employed convenience sampling where we identified all the existing WhatsApp groups of students at the different campuses of the university through a coordinator for each specific campus. The Google Form link to the questionnaire was then sent to the potential participants via the identified WhatsApp groups.

#### Qualitative Data

For the qualitative analysis, randomly identified students from the university database were contacted via phone call for consent to be interviewed after which a convenient time was scheduled for a recorded interview over Zoom video communications Inc. and via phone call. Participants were recruited until we reached a saturation point for the responses being given by participants

### Study Variables

Independent variables were demographic characteristics like sex, age, program offered and dependent variables included anxiety levels, challenges and coping strategies used by students.

### Data Management Analysis

#### Quantitative data analysis

The completed questionnaires were extracted from Google Forms and exported to a Microsoft Excel 2016 for cleaning and coding. The cleaned data was exported to STATA version 16.0 for analysis. Numerical data is summarized as means and standard deviations as appropriate. Categorical data is summarized as frequencies and proportions. Associations between independent variables and dependent variables were assessed using chi-square test or Fisher’s exact test and multivariate logistic regression analysis. A P < .05 was considered statistically significant.

#### Qualitative data analysis

All the responses from the participants were analyzed and reported as one dataset. The audio-taped students’ responses were transcribed and analyzed by qualitative content analysis as described by Granehelm and Lundman [22]. This included first reading the responses on the scripts to obtain an understanding of their contents and then meaning units were identified. The meaning units were then coded according to their content and these codes were grouped into categories. Summarizing the data, coding and development of categories were performed by the authors using thematic analysis.

### Ethical considerations

Prior to collecting data, we sought ethical clearance from Mbale Regional Referral Hospital Research Ethics Committee (MRRHREC) approval number MRRHREC 2020-02-05. The students were informed that participation in the study was voluntary and an electronic informed consent was sought on the initial page of the questionnaire. The ethical principles of involvement of human research subjects as outlined in the Nuremberg code and in the Declaration of Helsinki were strictly adhered to.

## Results

### Socio-demographic Characteristics of participants

A total of 338 students completed the survey and their median age was 23 years within the interquartile range of 21 to 25 years. Majority were male (n=213, 63%), undergraduates (n=288, 85%), single (n=297, 88%), and in their second year of study (n=113, 33%). The faculty of health sciences had the highest number (n=153, 45%) of participants (Table 1).

### Prevalence of Anxiety and associated factors among students

**Figure 1:**
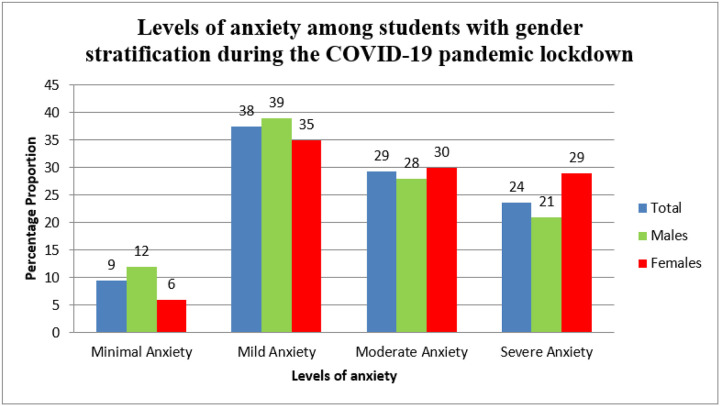
Levels of anxiety among students

Overall, 53% (n=179) of the students had anxiety. Of those with anxiety, a majority were males (n=105, 59%), in the age group of 18–25 years (n=153, 85%), staying at home during the lockdown (n=107, 60%), and from the faculty of health sciences (n=69, 39%). Majority of the students (n=237, 70%) were most concerned about graduating on time (Table 1). Most students with anxiety had mild form (38%, n=127). Females had higher levels of severe anxiety (n=36, 29%) and moderate anxiety (n=38, 30%) compared to males (Figure 1).

On bivariate analysis, marital status (p=0.024), worry inadequate internet facilities to support remote learning (p=0.000), uncertainty of how assessments would be done (p=0.004), completion of the semester on time (p=0.001), and getting a job after school (p=0.000) were significantly associated with anxiety (Table 1).

**Figure 2:**
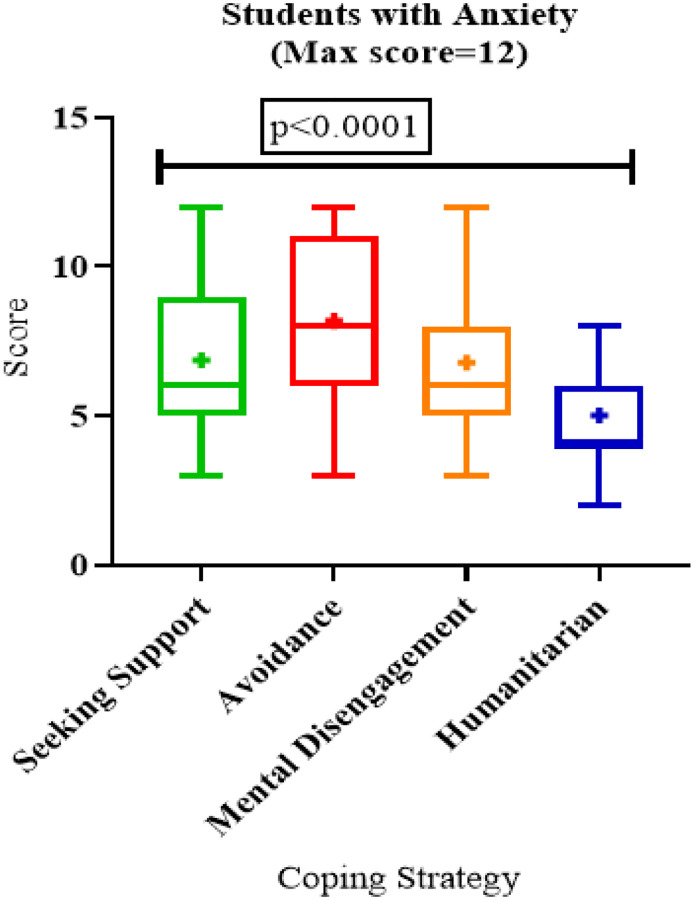
Coping Strategies among students with anxiety during the COVID-19 pandemic

On multivariate analysis, students concerned about inadequate internet facilities were twice more likely to have anxiety (aOR 2.0, 95% CI 1.1–3.7; p=0.021). There was no significant association of anxiety among students with sex, age, their year of study, faculty, or coping strategy (Table 2). Among those with anxiety, avoidance coping strategies had higher scores with a median of 8 (3–12) compared to other strategies (p<0.001) (Figure 2).

#### THEMATIC ANALYSIS OF THE CHALLENGES FACED BY STUDENTS DURING THE COVID-19 PANDEMIC LOCKDOWN

The in-depth interview revealed five main themes related to challenges faced by students during the lock down Figure 4.

##### Theme 1: Difficulties of online Learning

###### Subtheme: Prohibitive data costs.

Most of the students were experiencing hardships to purchase data bundles to enable them attend classes online. The lockdown affected economy as well and majority of these students are dependent on their parents for financial support who were also not probably working. One of the students elaborated on how expensive it is to meet these costs.

“I use my little money to buy data and (…) a lecture going on for 3 hours that’s like 1GB which is 4,000 UGX [approx. 1 USD] and if you have 5 lectures, that is 20,000 UGX [about 5 USD], which is a bit expensive.”

(STUD-F, Female student at Faculty of Engineering)

###### Subtheme: Poor and erratic internet connectivity.

The majority of the students were resident in the rural areas with poor internet connections that made smooth attendance of lectures almost impossible. This stressed out the students as they would miss important explanations of concepts from the lecturers.

“My network was really bad; literally you are in and out of the lecture and miss a lot of things”

(STUD-V, Female student at Faculty of Health Sciences)

###### Subtheme: Unreliable electric power supply.

In addition to the poor network, the situation was compounded by challenges of electric power connectivity for most students residing in the rural but also some urban areas. Some students totally stayed in areas dependent on solar power and others had unstable supply.

“The gadgets we use depend on power and we have power shortages and its pressing. There was a situation where I had 5% battery and the phone couldn’t go on zoom so I ended up missing the lecture”

(STUD-Q, Male student at Faculty of Management Science)

###### Subtheme: Using the Learning management system (LMS).

There were also reports of difficulties with access and navigation of the online learning management system (LMS) that was introduced by the university to ensure continuity of learning while at home.

“(…) the LMS system they introduced is not working to some of us and so for me I am not studying”

(STUD-B, Male student at Faculty of natural resources and environmental sciences)

###### Subtheme: Commitment of Lecturers.

Furthermore to constrain learning, limited engagement with lecturers was another concern put across by most students. They noted a lack of commitment from the lecturers to teach as some would continuously miss their scheduled online lectures.

“(…) the e-learning system they gave us is not reliable. The lectures are not consistent (…).”

(STUD-B, Female student at Faculty of Agriculture and animal sciences)

##### Theme 2: Delayed Academic Progress

###### Subtheme: Timeliness of completing the programs.

This was an area of apprehension for many students concerned of when they would finish their studies and move on to other ventures of life. The consecutive lockdowns made them lose hope on when the learning would become stable and continuous without interruptions.

“The course was supposed to take only two years but now I feel like it may even go up to 4 years, I am even fed up of studying.

(STUD-Y, Female student at Faculty of Agriculture and animal sciences)

###### Subtheme: Uncertainty about resumption of school.

Many students were not only worried about when they will finish but also the fact that they may never resume learning. They expressed growing concerns about when they would be able to return to school and finish their respective courses.

“It is really hard, you find that you are at home today, the other day, another day and we are not even sure when we shall go back to school, I keep asking myself, Shall we really go back to school?”

(STUD-A, Male student at Faculty of natural resources and environmental sciences)

###### Subtheme: Accumulating workload.

As well majority of the students were overwhelmed by the increasing workload and difficulty balancing academics, home and work responsibilities. Full learning wouldn’t continue entirely online especially for practical programs therefore many course units were left hanging before progressing into others. Like one student elaborated:

“We started semester 2 without doing examinations for semester 1 and that was another blow knowing you have all the 4 course units on your mind and still adding on others”

(STUD-V, Female student at Faculty of health sciences)

##### Theme 3: Fear of COVID-19 vaccination

Some participants reported fear of taking the vaccine due to their uncertainties and unreliability mostly caused by insufficient knowledge on safety of the vaccine and spreading “infodemic” about the vaccine’s safety and effectiveness. As majority of the students relied on social media for their information during lockdown, this was an inevitable happening. One of the students vividly expressed their fears of the vaccine:

“The vaccine is so dangerous according to me. I am so scared about the vaccine, because I hear in the news, first of all when you receive the vaccine when you have the virus you die. If you get vaccine when you have some other diseases with in you, you might die or have some other complications”

(STUD-E, Female student at Faculty of Management sciences)

##### Theme 4: Contracting COVID-19

The fear of COVID-19 ravaging the globe didn’t spare majority of the students as well. There was heightened fear among almost all students of either them or their loved ones contracting COVID-19 as many deaths were being recorded and broadcasted over news every other day. The fear was even heightened among those that experienced COVID-19 related symptoms like one student narrated:

“I was sick with cough, flue and back pain. I had everything, I was down, but I didn’t go for the COVID-19 test. The person I was staying with, after sometime got the same symptoms and for her, she went for the test and she tested Positive. I felt bad, I felt like I was going to die (…) I was seeing people dying and even the neighbor had passed on because of COVID-19”

(STUD-A, Female student at Faculty of natural resources and environmental sciences)

##### Theme 5: Changes to daily routine

Almost all students experienced changes in one aspect of their daily routines and lifestyles. They mentioned having realised changes to their appetite as well as eating patterns and increased period of sleep time as a consequence of the various fears and worries as a result of the COVID-19 induced lockdown.

“I may go a day without eating because of the stress. I am the one who cooks but I even fail to eat the food”.

(STUD-H, Female student at Faculty of Engineering)

“(…) I am finding a challenge of reading and concentrating on books while at home.

(STUD-AA, Female student at Faculty of Health Sciences)

#### THE COPING STRATEGIES USED BY STUDENTS DURING THE COVID-19 PANDEMIC LOCKDOWN

To contain the stress all students employed a coping strategy to avoid break down. From our interactions, several coping strategies to the lockdown induced stress and anxiety were identified from the responses of the study participants.

##### Theme 1: Support seeking

The respondents acknowledged seeking out family and social support whenever they felt overwhelmed to share their experiences, get guidance or counselling to go through whatever situation was pressing at the time. The common refuge was parents and friends for all the respondents.

“(…) basically I talk to someone, my big brother, or my mum or call a friend…”

(STUD-R, Female student at Faculty of Agriculture and Animal Sciences)

##### Theme 2: Relaxation

The other coping measure employed was relaxation. The measures included; watching movies/TV, listening to music, chatting with a friend(s) physically or online via social media, reading novels, playing games, exercising and taking a walk.

“I go and watch a movie, play games (…) I try so hard to engage in craft work, keep busy, do some exercise (…) I reach out to some friends over calls and WhatsApp video call but some are offline and we can’t reach them ”

(STUD-J, Female student at Faculty of Science and Education)

##### Theme 3: Problem solving

Facing the problem head-on and coming up with solutions to deal with a problem or anxiety was another coping strategy. Some respondents did something about their situation and sought solutions to whatever problem they faced.

“I have a project I am doing about fish farming so I spend most of my time at my fish pond, I don’t go anywhere and that helps me not to think about COVID-19”

(STUD-S, Female student at Faculty of Agriculture and Animal Science)

##### Theme 4: Escape/avoidance

Some students opted to stay away from news, events or situations that added on their stress or which they thought increased their risk of contracting or transmitting COVID-19. These included avoiding CoVID-19 related news updates and staying away from social gatherings. However, none of the respondents engaged in self-harm behavior though there were unhealthy coping behaviors reported.

“I have tried as much as I can to stay away from updates concerning COVID-19 (…) I don’t want anything to do with COVID updates”

(STUD-D, Female student at Faculty of Natural resources and environmental sciences).

## Discussion

COVID-19 continues to claim lives of thousands globally significantly creating mental stress among the general population [23]. This has been reported as well during previous infectious disease outbreaks like SARS, MERS, Ebola, the 2009 and 2010 H1N1 influenza pandemic [24–26] however the fear of COVID-19 is much greater. Students in higher institutions of learning like universities are vulnerable to higher levels of psychological distress including stress, anxiety and depression due to unique external stressors both academic and socioeconomic [7,8,15].

At such times of a pandemic ravaging the entire globe, with public health restrictions and closure of schools, the students face additional stressors especially in developing countries like Uganda where distance online learning is not well established and the continuity of learning faces inevitable difficulties. This is likely to increase their levels of anxiety. The present study provides information on the levels of anxiety, challenges and coping strategies employed by students during the second COVID-19 pandemic lock down at a university in Uganda.

In this study, more than half of the students (53%) experienced anxiety and mostly mild anxiety. Although anxiety was higher among the males (59%) more females experienced severe and moderate anxiety in agreement with different studies that have reported females to suffer from severe forms of anxiety [14,27,28]. Students in the age group of 18–25 years had the highest (85%) level of anxiety corresponding to other studies that have reported higher levels of anxiety in younger people [27,29]. As Solomou et al put it this negative correlation of age and anxiety could be explained by tenacity built along the years as we get exposed to multiple stressors [27]. The highest number of students (39%) experiencing anxiety was from the faculty health sciences (39%) and engineering (23%) in consonance with several other studies [14,30,31]. This is possibly due to the heavy workloads, higher academic expectations and frequent examinations and tests among other things.

Compared to other studies that reported differences in levels of psychological distress among students in different classes [15,32] our study showed no statistically significant difference. This is possibly because all students irrespective of class were facing the same situation of being home amidst closed schools. Additionally, our study showed no significant correlation between source of information about COVID-19 and anxiety unlike other studies that have reported a positive correlation between use of social media and anxiety during the pandemic among university student [33,34]. Students who were worried about inadequate internet facilities were twice more likely to have anxiety (aOR 2.0, 95% CI 1.1–3.7; p = 0.021). Since learning has shifted online it’s reasonable that those without Internet facilities would be apprehensive of how they will catch up with the studying. Worrying about how assessments would be done (p = 0.004), completion of the semester on time (p = 0.001), and getting a job after school (p = 0.000) were significantly associated with anxiety. This is similar to Chinese students who were also worried about online learning and when their new terms would proceed [14].

In Qualitative analysis students used a variety of coping strategies ranging from seeking support, relaxing to avoidance. Although quantitatively, univariate results showed that avoidance coping was most employed, qualitatively more of seeking support was reported by participants. Students with anxiety were more likely to employ the avoidance than other coping strategies. This is similar to findings reported by Nurunnabi and colleagues [19] among Chinese students. Both studies report findings at a point in time during a deadly wave of COVID-19 in either countries therefore it’s reasonable that students had to employ possible strategies there is to curb their anxiety.

### Strength and Limitations

This was a mixed method study and, per our knowledge, is the first of its kind to provide both quantitative and qualitative data about anxiety, challenges and coping strategies among students in a university due to the ongoing lockdown in Uganda. However, our study has some limitations. Firstly, this is a cross-sectional study done at only one university in Uganda with a small sample size. A bigger survey involving different universities could be conducted to conveniently generalize the findings. Secondly, we used a convenient online survey, which may contribute to some bias in the study results.

## Conclusion

The study provides insight into the anxiety levels of students and its severity among the different categories that the university authority should look out for. The largest number of students had anxiety especially those from faculty of health sciences and engineering of which most used avoidance strategies to cope up with the anxiety. This sheds light onto the different coping strategies that students employ which may not be used only during the pandemic but also stressful situations in the course of learning. This should be used to design appropriate strategies to help students cope in a better way to preserve their mental health.

## Figures and Tables

**Figure 3:**
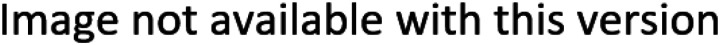
Figures 1, 3, and 4 not available with this version.

**Table 1:** Sociodemographic characteristics and factors associated with anxiety among students during the COVID-19 pandemic lockdown (N=338)

VARIABLE	N (%)	ANXIETY		P-VALUE
		YES N (%)	NO N (%)	
**All participants**		179 (53)	159 (47)	
**Age (Median, range)**	23 (21–25)			
18–25	276 (82)	153 (85)	123 (77)	0.096
26–35	55 (16)	22 (13)	33 (21)	
36 and above	7 (2)	4 (2)	3 (2)	
**Gender**				
Female	125 (37)	74 (41)	51 (32)	0.078
Male	213 (63)	105 (59)	108 (68)	
**Level of Study**				
Certificate	26 (8)	14 (8)	12 (8)	0.583
Diploma	18 (5)	12 (7)	6 (4)	
Undergraduate	288 (85)	149 (83)	139 (87)	
Postgraduate	6 (2)	4 (2)	2 (1)	
**Year of Study**				
Year 1	91 (27)	49 (27)	42 (26)	0.360
Year 2	113 (33)	65 (36)	48 (30)	
Year 3	56 (17)	31 (17)	25 (16)	
Year 4	40 (12)	19 (11)	21 (13)	
Year 5	38 (11)	15 (8)	23 (14)	
**Faculty**				
Health Sciences	153 (45)	69 (39)	84 (53)	**0.166**
Management Sciences	25 (7)	14 (8)	11 (7)	
Engineering	71 (21)	41 (23)	30 (19)	
Natural resources and environmental sciences	23 (7)	14 (8)	9 (6)	
Agriculture and Animal sciences	44 (13)	26 (15)	18 (11)	
Education sciences	22 (7)	15 (8)	7 (4)	
**Residence**				
University Hall	18 (5)	12 (6)	6 (4)	0.456
Rented Room	118 (35)	60 (34)	58 (36)	
Home	202 (60)	107 (60)	95 (60)52	
**Marital Status**				
Single	297 (88)	161 (90)	136 (86)	**0.024**
Married	37 (11)	14 (8)	23 (14)	
Divorced	4 (1)	4 (2)	0 (0)	
**Person you live with**				
Alone	89 (26)	52 (29)	37 (23)	0.475
Family member	193 (57)	101 (56)	92 (58)	
Relatives	19 (5)	10 (6)	9 (6)	
Roommate	37 (11)	16 (9)	21 (13)	
**Source of information**				
Social media	191 (57)	94 (53)	97 (61)	0.355
Journal	11 (3)	4 (2)	7 (4)	
Newspaper	2 (1)	1 (1)	1 (1)	
Radio	46 (14)	27 (15	19 (12)	
Television	83 (25)	50 (28)	33 (21)	
YouTube	3 (1)	1 (1)	2 (1)	
**Concerns of Students**				
Coping with online classes	182 (54)	99 (55)	83 (52)	0.568
Inadequate internet facilities	212 (63)	129 (72)	83 (52)	**0.000**
Lecturers not competent in delivering class online	137 (41)	84 (47)	53 (33)	**0.011**
Not sure how assessments will be done	214 (63)	126 (70)	88 (55)	**0.004**
Not sure if I can complete this semester on time	225 (67)	134 (74)	91 (57)	**0.001**
Not sure if I will graduate on time	237 (70)	143 (80)	94 (59)	**0.000**
Not sure if I will get a job after graduation	126 (37)	86 (48)	40 (25)	**0.000**
Uncertainty about internship	181 (54)	115 (64)	66 (42)	**0.000**
Uncertainty in the academic semester/year	219 (65)	131 (73)	88 (55)	**0.001**
Not sure if school will ever progress again without more lockdown interruptions	212 (63)	131 (73)	81 (51)	**0.000**

**Table 2:** Multivariate Logistic Regression showing factors associated with anxiety among students during COVID-19 pandemic lockdown

VARIABLE	AOR (95% Cl)	P-VALUE
**Age (Median, range)**		
18–25	1.0	
26–35	0.6 (0.2–1.4)	0.270
36 and above	1.8 (0.2–12.6)	0.521
**Gender**		
Male	1.0	
Female	1.6 (0.9–2.6)	0.093
**Year of Study**		
Year 1	1.0	
Year 2	1.2 (0.6–2.5)	0.577
Year 3	1.1 (0.4–2.4)	0.874
Year 4	0.7 (0.3–1.8)	0.525
Year 5	1.0 (0.3–2.9)	0.924
**Faculty**		
Education sciences	1.0	
Management Sciences	0.8 (0.2–3.2)	0.820
Engineering	0.9 (0.2–3.0)	0.897
Natural and environmental sciences	1.5 (0.3–6.9)	0.594
Agriculture and Animal sciences	0.8 (0.2–2.8)	0.818
Health Science	0.4 (0.1–1.5)	0.215
**Marital Status**		
Single	1.0	
Married	0.9 (0.3–2.6)	0.888
Divorced	1.0 (1.1–3.6)	0.745
**Source of information**		
Newspaper	1.0	
Journal	1.0 (0.0–36.9)	0.996
Radio	1.6 (0.1–50.6)	0.774
Social media	1.3 (0.0–39.8)	0.846
Television	2.0 (0.1–60.1)	0.670
YouTube	0.5 (0.036.0)	0.779
**Concerns of Students**		
Coping with online classes	1.0	**0.021**
Inadequate internet facilities	2.0 (1.1–3.7)	0.747
Lecturers’ competence in delivering online	0.9 (0.5–1.6)	0.753
Not sure how assessments will be done	0.9 (0.4–1.7)	0.611
Not sure if I can complete this semester on time	1.2 (0.5–2.8)	0.291
Not sure if I will graduate on time	1.6 (0.6–3.9)	0.153
Not sure if I will get a job after graduation	1.5 (0.8–3.1)	0.540
Uncertainty about internship	1.2 (0.5–2.7)	0.509
Uncertainty in the academic semester/year	0.7 (0.3–1.7)	0.367
If school will ever progress again without more lockdown	1.3 (0.6–2.7)	

## Data Availability

The datasets used and/or analyzed during the current study are available from the corresponding author on reasonable request.

## Abbreviations

L.M.S: Learning Management System
ODEL: Open Distance Electronic-Learning
SARS: Severe Acute Respiratory Syndrome
MERS: Middle East Respiratory Syndrome
